# Effects of melatonin and transcranial photobiomodulation on neuroinflammation-related gene expression and anxiety-like behaviors in rats under chronic restraint stress

**DOI:** 10.1016/j.ibneur.2026.10.009

**Published:** 2026-10-18

**Authors:** Mohammad Mazaheri, Maryam Radahmadi, Fahimeh Hosseini-Nasab, Nahid Eskandari

**Affiliations:** a Applied Physiology Research Center, Cardiovascular Research Institute, Isfahan University of Medical Sciences, Isfahan, Iran; b Department of Physiology, School of Medicine, Isfahan University of Medical Sciences, Isfahan, Iran; c Department of Immunology, School of Medicine, Isfahan University of Medical Sciences, Isfahan, Iran

**Keywords:** Chronic stress, Neuroinflammation-related gene, Transcranial photobiomodulation, Melatonin, Anxiety-like behaviors

## Abstract

Psychological stress causes immunological and behavioral alterations that can maladaptively affect the quality of life. However, melatonin (MLT) and transcranial photobiomodulation (tPBM) have promising effects on brain functions. This study investigated the effects of MLT and tPBM in rats under chronic stress by evaluating neuroinflammation-related gene expression and anxiety-like behaviors. Sixty-three male Wistar rats were randomly assigned to nine groups: Control (Co), Sham injection (Sh-I), Sham tPBM (Sh-tPBM), MLT, tPBM, Stress (St), Stress with Melatonin (St-MLT), Stress with tPBM (St-tPBM), and Stress with Melatonin and tPBM (St-MLT-tPBM). Chronic restraint stress (2 h/day for 21 days) was applied to all stressed rats. MLT (20 mg/kg; intraperitoneal) and tPBM (808 nm; delivered to the scalp surface) were administered following restraint stress. Gene expression of hippocampal neuroinflammation-associated genes (IL-17A, ICAM-1, VCAM-1, IL-1β, and Aif1) was measured by real-time PCR. Additionally, anxiety-like behaviors were evaluated by the elevated-plus maze test. Hippocampal IL-17A and ICAM-1 gene expression was higher in stressed rats than in normal subjects. However, in the St group, IL-1β expression remained unchanged, whereas Aif1 expression was decreased compared with the Co group. tPBM reversed the stress-induced upregulation of IL-17A and ICAM-1 expression, whereas MLT could not mitigate them compared with normal subjects. The combined MLT and tPBM treatment elicited gene expression changes similar to those observed with tPBM alone. Stress-induced anxiety-like behaviors were significantly reduced by MLT, with effects similar to those of the combined treatment, which outperformed tPBM alone. These findings suggest the potential of tPBM and melatonin as complementary approaches for mitigating stress-related alterations, highlighting further investigation to optimize their doses and treatment parameters.

## 1 Introduction

Stress is generally defined as any life event or condition that disrupts the organism through real physiological or perceived psychological stressors. In response, the body initiates adaptive mechanisms to preserve well-being and maintain homeostasis (Kim and Kim, 2023a). Psychosocial stress is considered a key factor in predicting the development and progression of stress-related psychopathologies in humans (Su et al., 2022). It triggers the hypothalamic–pituitary–adrenal (HPA) axis, resulting in glucocorticoid release—cortisol in humans and corticosterone in rodents—from the adrenal glands, along with activation of the sympathoadrenal–medullary (SAM) system (Lupien et al., 2009, Zhu et al., 2019). Evidence from preclinical and clinical investigations indicates that psychological stress can hyperactivate immune pathways, increasing the production of pro-inflammatory cytokines and contributing to alterations in brain function and the development of maladaptive behaviors (Johnson et al., 2019, Hassamal, 2023b). Neuroinflammation is characterized by immune cell infiltration into the central nervous system (CNS) and activation of resident immune cells, including microglia and astrocytes (Shi and Yong, 2025). It is associated with mental disorders characterized by severe symptoms and increased morbidity and mortality (Maes et al., 2012). Immune cell infiltration across the blood–brain barrier (BBB) is mediated by interactions between endothelial cell adhesion molecules (CAMs) and their ligands on migrating immune cells (Fournier et al., 2022, Medina-Rodriguez and Beurel, 2022). In this process, leukocyte adhesion is facilitated by leukocyte integrins and by intercellular adhesion molecules (ICAMs) and vascular cell adhesion molecules (VCAMs) expressed on the endothelium (Wautier and Wautier, 2021). Endothelial ICAM-1 and VCAM-1 are important mediators of leukocyte adhesion and transmigration across the BBB (Speyer and Ward, 2011). Following infiltration into the brain, Th17 cells, a subset of CD4⁺ T helper cells, secrete the pro-inflammatory cytokine interleukin (IL)-17A, thereby promoting neuroinflammation (Kowalczyk et al., 2019, Shi et al., 2022b, Wang et al., 2024). IL-17A has been implicated in BBB endothelial activation and leukocyte recruitment, partly through modulation of CAMs and chemokine signaling (Guo et al., 2026, Maione et al., 2021, Wojkowska et al., 2017b). Recent studies suggest that the pro-inflammatory cytokine IL-17A contributes to the pathogenesis of inflammatory, autoimmune, and neuropsychiatric disorders (Nadeem et al., 2017, Kim et al., 2021). Severe psychological stress has been associated with changes in microglial morphology and inflammatory activity, including alterations in the expression of microglia-associated markers such as ionized calcium-binding adapter molecule 1 (Iba1)(Schramm and Waisman, 2022, Catale et al., 2020, Tian et al., 2023, Ransohoff, 2016). Furthermore, they increase the expression of ionized calcium-binding adapter molecule 1 (Iba1) (Schramm and Waisman, 2022). Iba1, encoded by the allograft inflammatory factor 1 (Aif1) gene, is an actin-binding protein expressed in microglia. Due to its diverse functions, Aif1 has been associated with macrophage activation and with several diseases, including neurological disorders (De Leon-Oliva et al., 2023). It has been proposed that Aif1 upregulation contributes to microglial activation through its role in remodeling the cytoskeleton and membrane structure (Réus et al., 2019, Galvani et al., 2021). Overactivation of microglia is also associated with increased release of major pro-inflammatory mediators, such as interleukin (IL)-1β, superoxide, nitric oxide, reactive oxygen species, and proteases (Zhang et al., 2019, Taoro-González et al., 2019, Stein et al., 2017). IL-1β is a pro-inflammatory cytokine regulated in part by the NF-κB pathway and, at physiological levels, contributes to normal brain function, including cognitive processes. However, when its levels are elevated, IL-1β can exert detrimental effects, including excitotoxicity and disruption of normal neuronal activity (White et al., 2024). Moreover, IL-17A and IL-1β are considered fundamental to sustaining inflammatory responses (Liu et al., 2018). One of the brain regions highly sensitive to stress hormones and particularly vulnerable to neuroinflammation induced by chronic stress is the hippocampus (White et al., 2024). It plays a central role in cognitive processes and is also involved in the regulation of stress, emotion, and affect (Fanselow and Dong, 2010). In addition, the hippocampus plays a key role in glucocorticoid-mediated negative feedback regulation of the HPA axis, helping to limit excessive release of stress hormones (Kim and Kim, 2023b).

Anxiety disorders are among the most common neuropsychiatric conditions worldwide (Javaid et al., 2023). Psychological stress and anxiety have intertwined behavioral and neural underpinnings (Daviu et al., 2019, Salpekar et al., 2020). The pathophysiology of anxiety is multifactorial, involving dysregulation of stress hormone secretion, increased proinflammatory cytokine signaling, alterations in neural signaling pathways, and increased oxidative stress (Repova et al., 2022, Liang et al., 2025). The hippocampus is a key brain region implicated in stress-related psychopathologies, including anxiety disorders (Shi et al., 2023). Current treatment strategies for anxiety disorders encompass a range of approaches, including psychotherapy, neuromodulation techniques, and pharmacological interventions (Tafet and Nemeroff, 2020, Guo et al., 2023).

Transcranial photobiomodulation (tPBM), also referred to as low-level laser therapy, is a non-invasive neuromodulation technique that uses low-intensity red to near-infrared (NIR) light and is considered safe and well-tolerated (Nairuz and Lee, 2024, Montazeri et al., 2021). Both coherent laser sources and incoherent light-emitting diodes (LEDs) are commonly applied in PBM therapy (Ma et al., 2024). Growing evidence suggests that tPBM can enhance brain function and behavioral outcomes in various neurological and psychological disorders through modulation of multiple cellular and molecular pathways (Abijo et al., 2023, Lee et al., 2023). Among these mechanisms, attenuation of neuroinflammation appears to play a critical role in improving neuropsychiatric conditions (Cardoso et al., 2022, Hamblin, 2017). During photon energy delivery, several physiological changes occur in the brain, including increased cerebral blood flow, greater oxygen availability and utilization, elevated adenosine triphosphate (ATP) production, and enhanced mitochondrial function (You et al., 2021, Abijo et al., 2023).

Melatonin (N-acetyl-5-methoxytryptamine, MLT) is a major neurohormone primarily secreted by the pineal gland (Fu et al., 2016). The pineal organ synthesizes melatonin predominantly at night and releases it into the systemic circulation as well as the cerebrospinal fluid (Pfeffer et al., 2022). Endogenous melatonin secretion is tightly regulated by the circadian rhythm and the light–dark cycle (Halabian and Radahmadi, 2024). In addition to its physiological role, exogenous melatonin has been reported to improve certain aspects of cognitive function in adults (Zakaria et al., 2016). The neuroprotective properties of MLT are largely attributed to its ability to reduce oxidative stress and attenuate neuroinflammation (Tyagi et al., 2010).

Research from preclinical and clinical studies indicates that the combination of tPBM therapy with additional treatment approaches can impact the treatment outcomes of various disorders (Farazi et al., 2024). In this regard, the current study was designed to investigate how MLT and tPBM could alter the potential inflammatory effects of chronic stress in the hippocampus at the gene expression level. In addition, whether each intervention can attenuate neuroinflammation by probable alterations in gene expression levels of mediators related to neuroinflammation (IL-1β, Aif1, IL-17A, ICAM-1, and VCAM-1), or through a combination of both mechanisms is also assessed at the gene expression level. Furthermore, anxiety-like behaviors, a key functional outcome linked to neuroinflammation (Won and Kim, 2020), were assessed to elucidate how possible anti-inflammatory effects of MLT and tPBM may affect behavioral phenotypes. Given the well-documented adverse effects and variable therapeutic efficacy of currently available antidepressants (Ramic et al., 2020), exploring alternative or adjunctive strategies with potentially favorable safety profiles, such as MLT and tPBM, appears justified.

## 2 Methods

Sixty-three male Wistar rats, with weights ranging from 200 g to 250 g, were utilized in the experiments. These rats were kept in identical cages under controlled environmental conditions, maintaining 50 ± 5% humidity, a temperature of 23 ± 2° C, and a 12/12-h light/dark cycle (lights on at 07:00). All experiments were approved by the Research Ethics Committee at Isfahan University of Medical Sciences (IR.MUI.AEC.1403.065). The animals were randomly assigned to nine groups. For animal allocation, predefined inclusion criteria were considered, including the specified body-weight range and the absence of apparent signs of disease or other health abnormalities (Percie Du Sert et al., 2020). During allocation to the experimental groups, we attempted to ensure an equal probability of receiving any particular treatment, without preferential selection based on observable characteristics or experimental condition, while maintaining balanced group sizes of seven animals per group, as follows:

• Control (Co) group: Rats were kept in their home cages without any treatment
• Sham Injection (Sh-I) group: Rats received injections of saline in equivalent volumes.
• Sham tPBM (Sh-tPBM) group: Rats were subjected to the same immobilization procedure as the tPBM group, but the laser was turned off.
• Melatonin (MLT) group: Rats received daily injections of melatonin at 20 mg/kg.
• Transcranial Photobiomodulation (tPBM) group: Rats were treated with tPBM at 192.2 J/cm² per session.
• Stress (St) group: Rats were exposed to chronic stress without any treatment.
• Stress-Melatonin (St-MLT) group: Stressed rats received daily melatonin at 20 mg/kg.
• Stress-tPBM (St-tPBM) group: Stressed rats received tPBM at 192.2 J/cm² per session.
• Stress-Melatonin-tPBM (St-MLT-tPBM) group: Stressed rats received both daily melatonin (20 mg/kg) and tPBM (192.2 J/cm² per session).

In the non-stress groups, interventions were performed between 10:00 and 12:00 a.m. Specifically, melatonin was administered to the MLT group, and tPBM therapy was applied to the tPBM group during this time window. In the stress groups, rats were exposed to the stress protocol daily from 8:00–10:00 a.m. In the St-MLT group, MLT was administered 30 min after the termination of the stress session. In the St-tPBM group, tPBM was applied at least 60 min following the end of the stress exposure. In the St-MLT-tPBM group, MLT was first administered 30 min after stress cessation, and tPBM was subsequently applied 30 min after melatonin injection (Fig. 1).

**Fig. 1:**
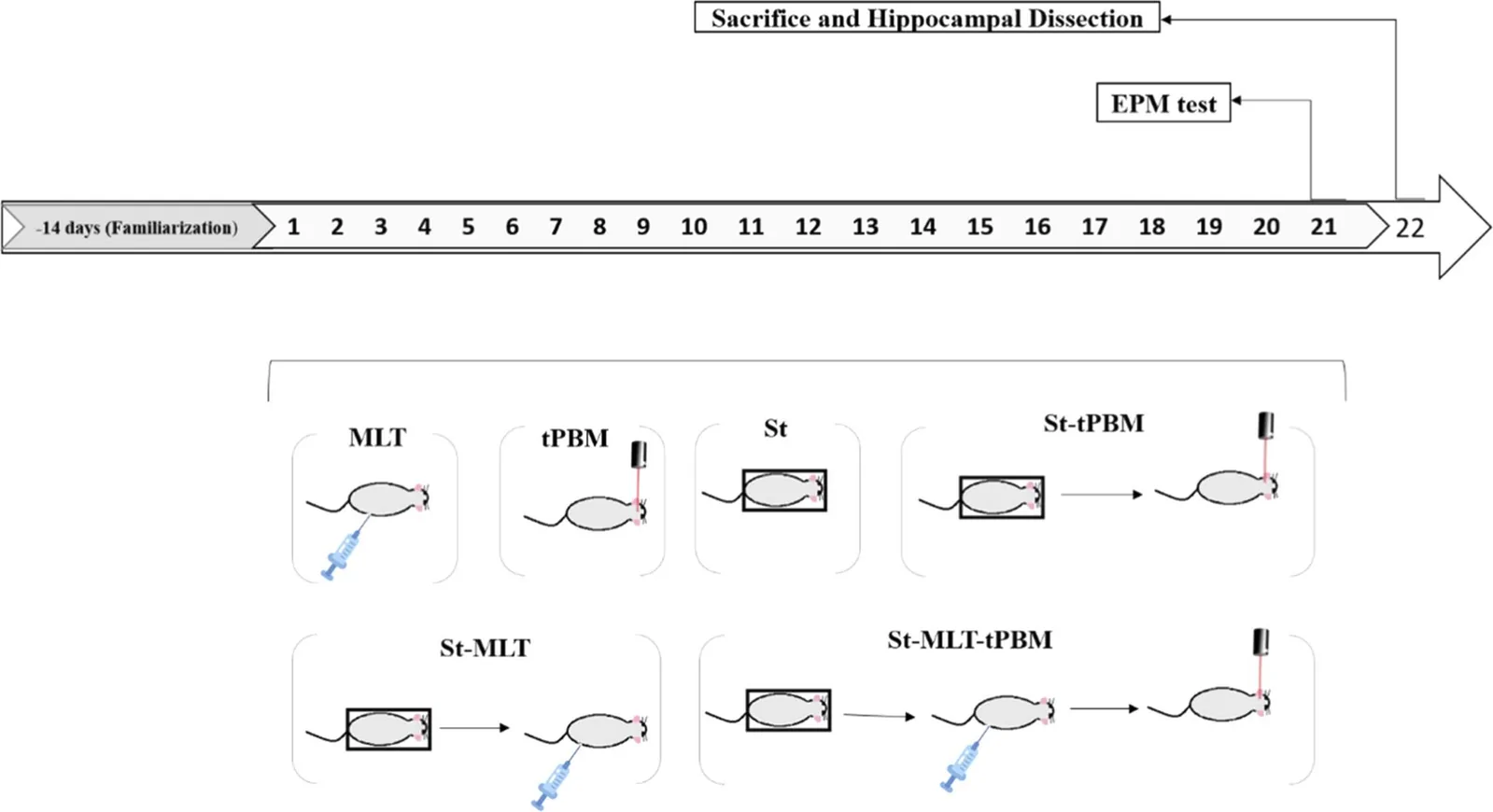
Experimental timeline and study design. After a 14-day familiarization period (day −14), animals were allocated to 9 groups. Chronic restraint stress was applied from day 1 to day 21. MLT and/or tPBM were administered according to group allocation. The elevated plus maze (EPM) test was performed on day 21, followed by sacrifice and hippocampal dissection on day 22.

### 2.1 Chronic stress paradigm

Animal (rat) modeling can yield significant experimental findings in physiological, psychological, and biological studies. Before initiating the experimental procedures, all animals underwent a 2-week familiarization period by being housed together in groups of four in each cage. In the present study, Stress induction was performed in identical-sized restraint cages (42 × 27 × 15 cm; Tajhiz Gostar Omid Iranian Co., Tehran, Iran). Chronic restraint stress is a preclinical model of psychological stress that is widely used in rodents (McGivern et al., 2009, Mazaheri et al., 2025a). It was induced (2 h/day, 08:00–10:00) for 21 consecutive days. Animals were monitored during the restraint sessions, and any animal meeting predefined endpoint criteria, such as respiratory distress, would have been removed from the experiment. It was applied by placing the rats in restrainers, after which each restrained rat was kept in an individual cage. Individual housing during restraint was used to minimize interference from social interactions (Mazaheri et al., 2025b, Mazaheri et al., 2024b) and to ensure that the restraint-induced stress was applied consistently.

### 2.2 Melatonin administration

After dissolving a 20 mg/kg dose of pure Melatonin powder (Amin Chemical & Pharmaceutical Co., Isfahan, Iran) in sterile 0.9% saline, the solution was administered intraperitoneally (i.p.) to both stressed and non-stressed rats for 21 consecutive days. Briefly, 20 mg of pure melatonin powder was dissolved in 1 cc of saline (Souza et al., 2014). The melatonin dose (20 mg/kg, i.p.) was selected based on previous studies using this dose in rodent models of chronic restraint stress and stress-related brain alterations (Fatima et al., 2020, Madhu et al., 2021, Eşrefoğlu et al., 2014).

### 2.3 Transcranial photobiomodulation protocol

Animals from the tPBM groups were immobilized (using the same procedure as the sham-tPBM group) without anesthesia. They received treatment with an 808 nm GaAlAs diode laser at a power of 115 mW (Saba Lase Technologies Inc., Toronto, Canada). In this study, the laser beam was applied to two points on the shaved scalp, anatomically above the right and left hippocampus (Hong et al., 2022). The laser beam spot size and the power density were 0.126 cm^2^ and 0.91 w/cm^2^, respectively. The present study targeted a radiant exposure (fluence) of 5 J/cm² at each hippocampal level (right and left hippocampus) (Lu et al., 2017, Li et al., 2021). Accordingly, to determine the radiant exposure at the scalp surface and the corresponding irradiation time required to deliver the target fluence to the hippocampal region, light penetration through different tissue samples was assessed as described below using isolated rat samples of the skull covered by scalp and the cortex.

#### 2.3.1 Tissue light transmission assessment

Because the target irradiation areas were the right and left hippocampi, optical transmission through the main intervening tissues (scalp, skull, and cortex) was assessed. Seven rats were used for this assessment before the main experiments. Following deep anesthesia through an intraperitoneal injection of ketamine (100 mg/kg) and xylazine (10 mg/kg) (Sigma-Aldrich Chemical Co., St. Louis, MO, USA), the animals were sacrificed, and tissue samples were obtained from the anatomical regions directly overlying the hippocampus. A laser power meter (Gentec-EO, Quebec City, Canada) was used to measure the transmitted optical power. The optical power transmitted through blank optical glass was measured as the reference, and tissue transmission was calculated as the ratio of transmitted power through each tissue sample to the corresponding blank-glass measurement (Salehpour et al., 2018, Salehpour et al., 2016). The mean transmission ratios for the scalp/skull and cortex were 0.206 and 0.254, respectively.

#### 2.3.2 Radiant exposure and irradiation time calculation

The overall transmission ratio to the hippocampal level (scalp and skull transmittance × cortical transmittance) was calculated as the product of these two ratios (0.206 × 0.254 = 0.0523, approximately 5.2%). Thus, to account for attenuation by the scalp–skull and cortical tissues, the incident radiant exposure required at the scalp surface to achieve a target radiant exposure of 5 J/cm² at the hippocampal level was determined using the formula (target radiant exposure / overall transmission ratio), yielding 5 J/cm² / 0.052 = 96.1 J/cm² per point and 192.2 J/cm² per session for the two treatment points. Based on the applied power density of 0.91 W/cm², the corresponding irradiation time was calculated using the formula (radiant exposure/power density), yielding 96.1 J/cm² / 0.91 W/cm² = 105 s. An irradiation time of 105 s was used per therapy point. The total energy delivered per treatment point was calculated as power × irradiation time, yielding 0.115 W × 105 s = 12 J per treatment point, and 24 J per session for the two treatment points.

### 2.4 Elevated Plus Maze (EPM)

The Elevated Plus Maze (EPM) test is widely employed to evaluate stress levels, anxiety-related behaviors, and the anxiolytic effects of various treatments in rodents (Gaspar et al., 2023). In the current study, an EPM apparatus was constructed from black, opaque Plexiglas and elevated 70 cm above the ground. The apparatus comprised two open arms, each measuring 60 cm in length, 10 cm in width, and 1 cm in height, as well as two closed arms, each measuring 60 cm in length, 10 cm in width, and 30 cm in height. These arms extended from a central platform, which measured 10 cm in length and 10 cm in width (Radahmadi and Farahbakhsh, 2025).

On the 21st day, the animals were individually positioned at the center of the EPM apparatus, facing the open arms. Each animal was then allowed to freely navigate through various sections of the maze for 300 s. The number of open arm entries (OAEs) and the total time spent in the open arms (OAT) were recorded over a duration of 300 s, serving as criteria for assessing motor activity and anxiety in the EPM tests. EPM anxiety-related measures, particularly the percentage of open-arm entries and time spent in the open arms, are relatively less dependent on general locomotor activity than measures such as total arm entries. Moreover, the preferential avoidance of open arms is considered to reflect anxiety-related behavior rather than overall locomotor activity (Várkonyi et al., 2022, Espejo, 1997, Braun et al., 2011). The percentage of OAEs [OAE% = (OAE/Total entries to the open and closed arms) × 100] and the percentage of OAT [Oat% = (OAT/300) × 100] were calculated (Mazaheri et al., 2024a). A substantial enhancement in these two parameters indicates reduced anxiety in the animals. It is important to note that entering the open arm requires the animal to place all four legs within the open arm (Jokar et al., 2025). After each experiment, the entire apparatus was cleaned with 70% alcohol to prevent contamination in subsequent experiments. A digital camera positioned above the EPM apparatus was used to capture the animals’ movements and transmit the recorded images to a personal computer running Neurovision software (Iran).

### 2.5 Gene expression assessment

On the 22nd day of the experiment, the rats were given anesthesia through an intraperitoneal injection of ketamine (100 mg/kg) and xylazine (10 mg/kg) (Sigma-Aldrich Chemical Co., St. Louis, MO, USA) (Jokar et al., 2023). The animals were euthanized by decapitation between 16:00 and 18:00. The hippocampi were rapidly dissected on dry ice, and the right hippocampus was collected for gene expression analysis of IL-17A, IL-1β, ICAM-1, VCAM-1, and *Aif1* genes. Gene expression analysis was performed using real-time polymerase chain reaction (PCR).

Total RNA was extracted from hippocampal tissue using the Total RNA Extraction Kit (ParsTous, Mashhad, Iran) according to the manufacturer's protocols. Briefly, tissue samples were lysed with a chaotropic salt-containing buffer, and the released RNA was bound to a silica membrane. After several washing steps with an ethanol-containing buffer, purified RNA was eluted using RNase-free deionized distilled water (ddH₂O). RNA integrity was verified by agarose gel electrophoresis, and its concentration was measured at 260 nm using a NanoDrop spectrophotometer.

Complementary DNA (cDNA) was synthesized from 5 ng of total RNA using random hexamer primers and a cDNA synthesis kit (ParsTous, Mashhad, Iran) according to the manufacturer's instructions. Quantitative real-time PCR was carried out on a StepOnePlus Real-Time PCR System (Applied Biosystems) using Real Q Plus 2 × Master Mix Green with High ROX™ (Ampliqon) and the primers listed in Table 1. Primer specificity was assessed in silico using NCBI BLAST against reference sequences of Rattus norvegicus. qPCR reactions were performed using SYBR Green chemistry on Applied Biosystems StepOne real-time PCR systems. Cq values were determined using the instrument-specific analysis software with a consistent thresholding approach across samples. For each condition, three independent biological replicates were randomly selected from the seven right hippocampal samples available in each group, and each biological replicate was analyzed in three technical replicates. Amplification profiles were inspected for consistency across technical replicates. Undetermined Cq values were considered non-detects and were excluded from quantitative analysis. Reactions showing inconsistent amplification among technical replicates were reviewed as potential technical outliers and excluded only when supported by the predefined quality-control criteria. Melting curves for the analyzed samples are provided in the Supplementary Figure. GAPDH was selected as the endogenous reference gene based on its widespread use in rodent brain and hippocampal RT-qPCR studies and its reported stability under various experimental conditions (Schwarz et al., 2020, Lopes et al., 2013, Uppalapati et al., 2025). The amplification protocol consisted of an initial denaturation at 95°C for 1 min, followed by repeated cycles of denaturation at 95°C for 15 s and annealing/extension at 60°C for 60 s. We calculated relative gene expression using the 2^−ΔΔCt method after normalizing to GAPDH, and we expressed results as fold changes relative to the corresponding control group.

**Table 1:** Primer information for RT-qPCR.

Primer	Accession No.	Sequences (5′ to 3′)	Amplicon Size (bP)	Reference
IL-17A	NM_001106897.1	Forward: CTACCTCAACCGTTCCACT Reverse: TTCTCAGGCTCCCTCTTC	189	(Ding et al., 2015)
IL-1β	NM_031512.2	Forward: CTCGTGGGATGATGACGACC Reverse: AGGCCACAGGGATTTTGTCG	212	(Wang et al., 2022)
Aif1	NM_017196.3	Forward: GCCTCATCGTCATCTCCCCA Reverse: AGGAAGTGCTTGTTGATCCCA	142	(Doorn et al., 2015)
ICAM-1	XM_063264947.1	Forward: TCTTGCGAAGACGAGAACCTC Reverse: GCTCTGGGAACGAATACACAG	193	(Bafadam et al., 2021)
VCAM-1	NM_012889.2	Forward: AAGTGGAGGTCTACTCATTCC Reverse: GGTCAAAGGGGTACACATTAG	114	(Khodadadi et al., 2018)
GAPDH	NM_017008.4	Forward: GGGGCTCTCTGCTCCTCCC Reverse: CGGCCAAATCCGTTCACACCG	106	(Lopes et al., 2013)

## 3 Results

### 3.1 Hippocampal IL-17A gene expression

IL-17A gene expression was significantly higher in the St group than in the non-stressed groups (p < 0.001 for all). Among stress-exposed animals, tPBM, MLT, and their combinations significantly reduced IL-17A gene expression levels in the stressed animals compared to the St group (p < 0.001 for all comparisons). However, IL-17A gene expression remained significantly elevated in the St-MLT group compared with the Co, Sh-tPBM, and MLT groups (p < 0.05 for Co and MLT; p < 0.001 for Sh-tPBM). It significantly decreased in the St-MLT-tPBM and tPBM groups compared to the Sh-I group (p < 0.05 for both) (Fig. 2).

**Fig. 2:**
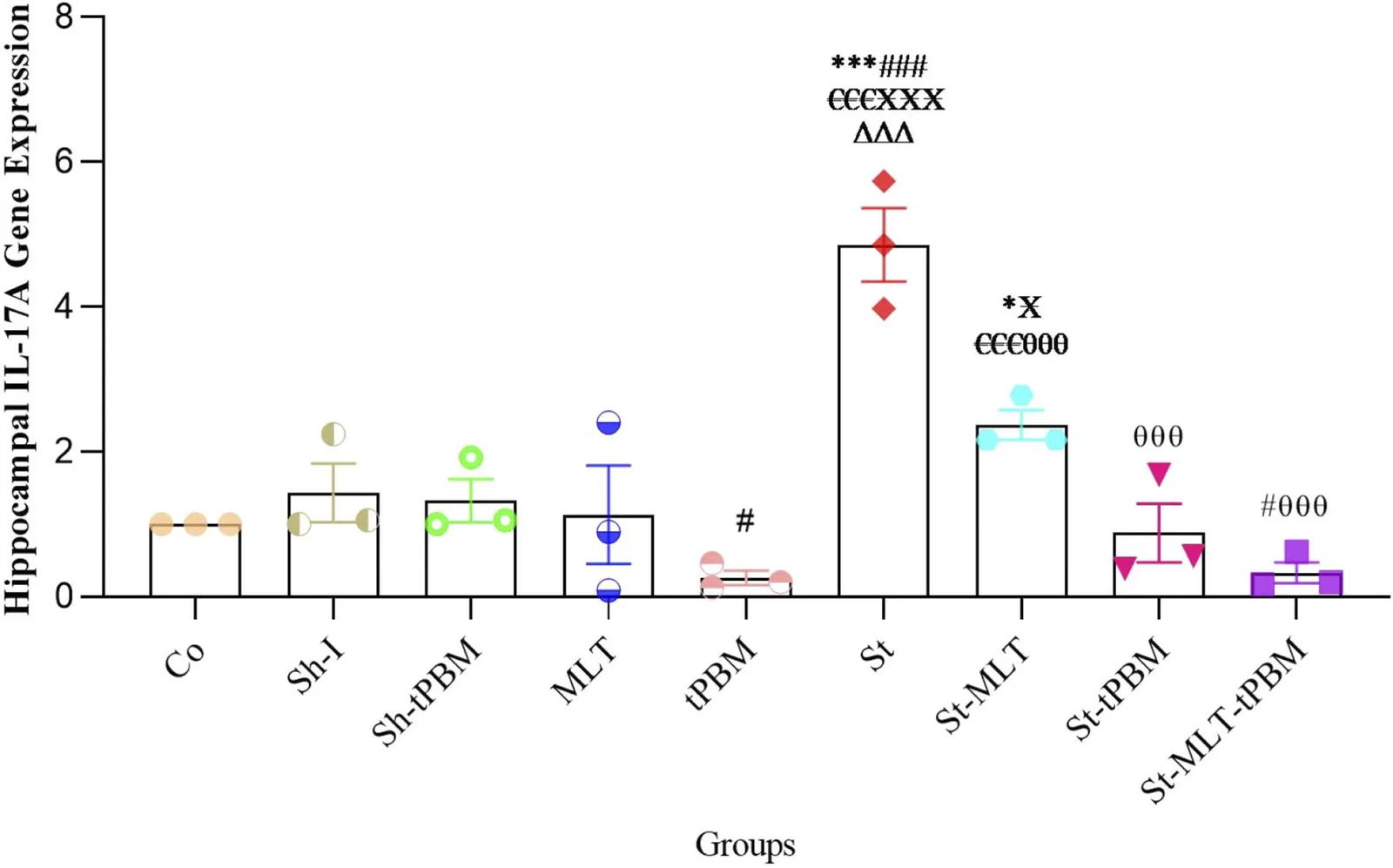
Hippocampal IL-17A gene expression measured by RT-qPCR (n = 3 independent biological replicates per group). The results are shown as the mean ± SEM (one-way ANOVA followed by LSD post hoc test). *P < 0.05 and ***P < 0.001 compared to the Co group. ^#^P < 0.05 and ^###^p < 0.001 compared to the Sh-I group. ^€€€^P < 0.001 compared to the Sh-tPBM. ^Ӿ^P < 0.05 and ^ӾӾӾ^p < 0.001 compared to the MLT group. ^ΔΔΔ^P^<^0.001 compared to the tPBM group. ^θθθ^P < 0.0001 compared to the St group.

### 3.2 Hippocampal ICAM-1 gene expression

As seen in Fig. 3, ICAM-1 gene expression was significantly increased in the St group compared to the Co and Sh-I groups (p < 0.05 for both comparisons). Conversely, it was significantly decreased in the St-tPBM group compared to the St, St-MLT, and tPBM groups (p < 0.05 for St and St-MLT; p < 0.01 for tPBM). In addition, ICAM-1 gene expression levels were significantly decreased with the co-administration of MLT and tPBM in the stressed samples compared to the St-MLT (p < 0.05) and tPBM (p < 0.01) groups. Nevertheless, it remained higher in the St-MLT group compared to the Co, Sh-I, and Sh-tPBM groups (p < 0.05 for all comparisons). tPBM therapy in the non-stressed animals increased ICAM- 1 gene expression levels significantly compared to the Co, Sh-I, Sh-tPBM, and MLT groups (p < 0.05 for MLT and p < 0.01 for all other comparisons).

**Fig. 3:**
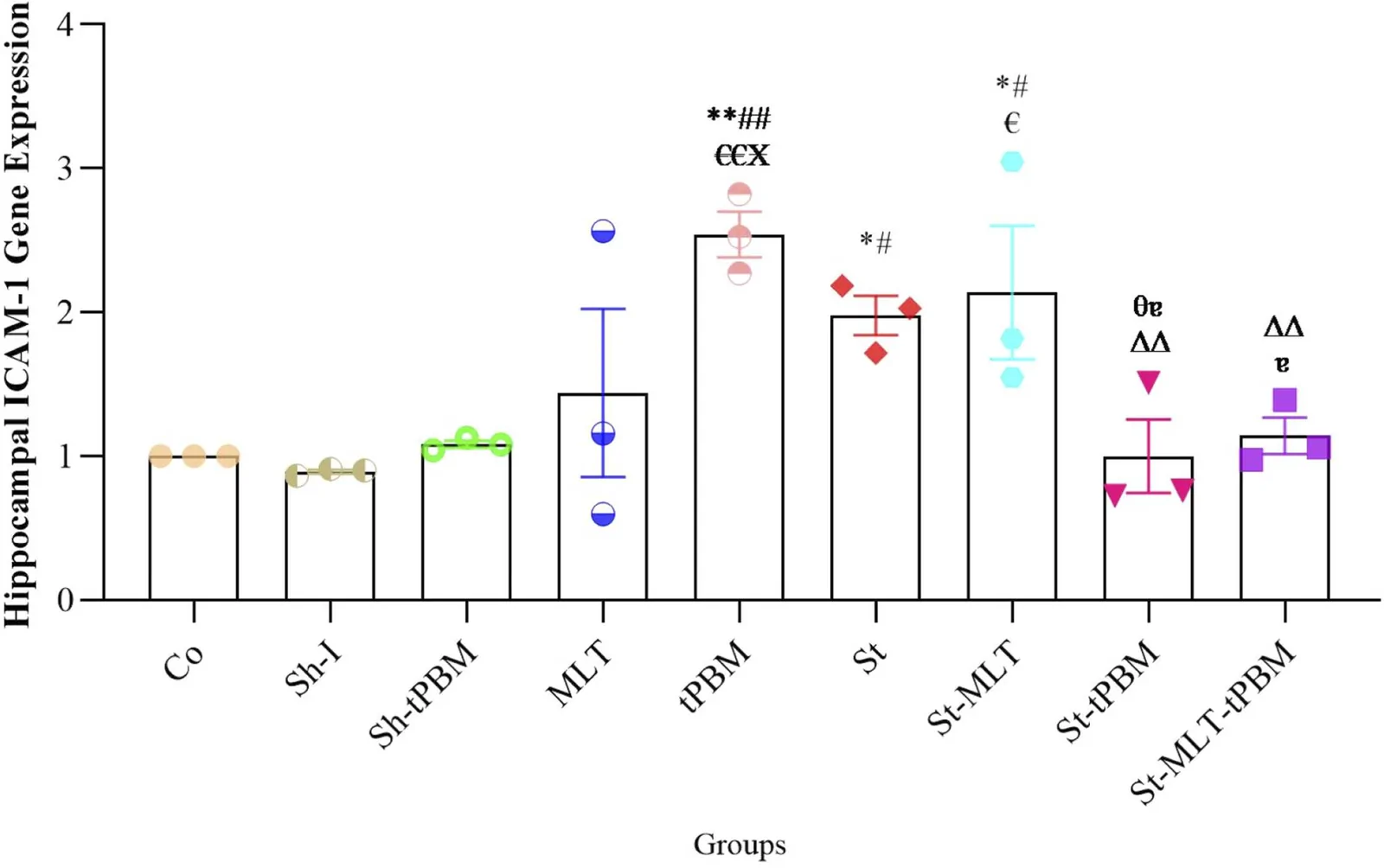
Hippocampal ICAM-1 gene expression measured by RT-PCR (n = 3 independent biological replicates per group). The results are shown as the mean ± SEM (one-way ANOVA followed by LSD post hoc test). *P < 0.05 and **P < 0.01 compared to the Co group. ^#^P < 0.05 and ^##^p < 0.01 compared to the Sh-I group. ^€^p < 0.05 and ^€€^P < 0.01 compared to the Sh-tPBM^. Ӿ^P < 0.05 compared to the MLT group. ^ΔΔ^P < 0.01 compared to the tPBM group. ^θ^P < 0.05 compared to the St group. ^ɐ^p < 0.05 compared to the St-MLT group.

### 3.3 Hippocampal VCAM-1 gene expression

Based on the data, hippocampal VCAM-1 gene expression was significantly increased in the St-MLT group compared with the Sh-I group (p < 0.05). VCAM-1 gene expression was also decreased in the St-tPBM (p < 0.05) and St-MLT-tPBM groups (p < 0.01) compared with the St-MLT group. Finally, it was significantly decreased in the St-MLT-tPBM group compared with the tPBM group (p < 0.05) (Fig. 4).

**Fig. 4:**
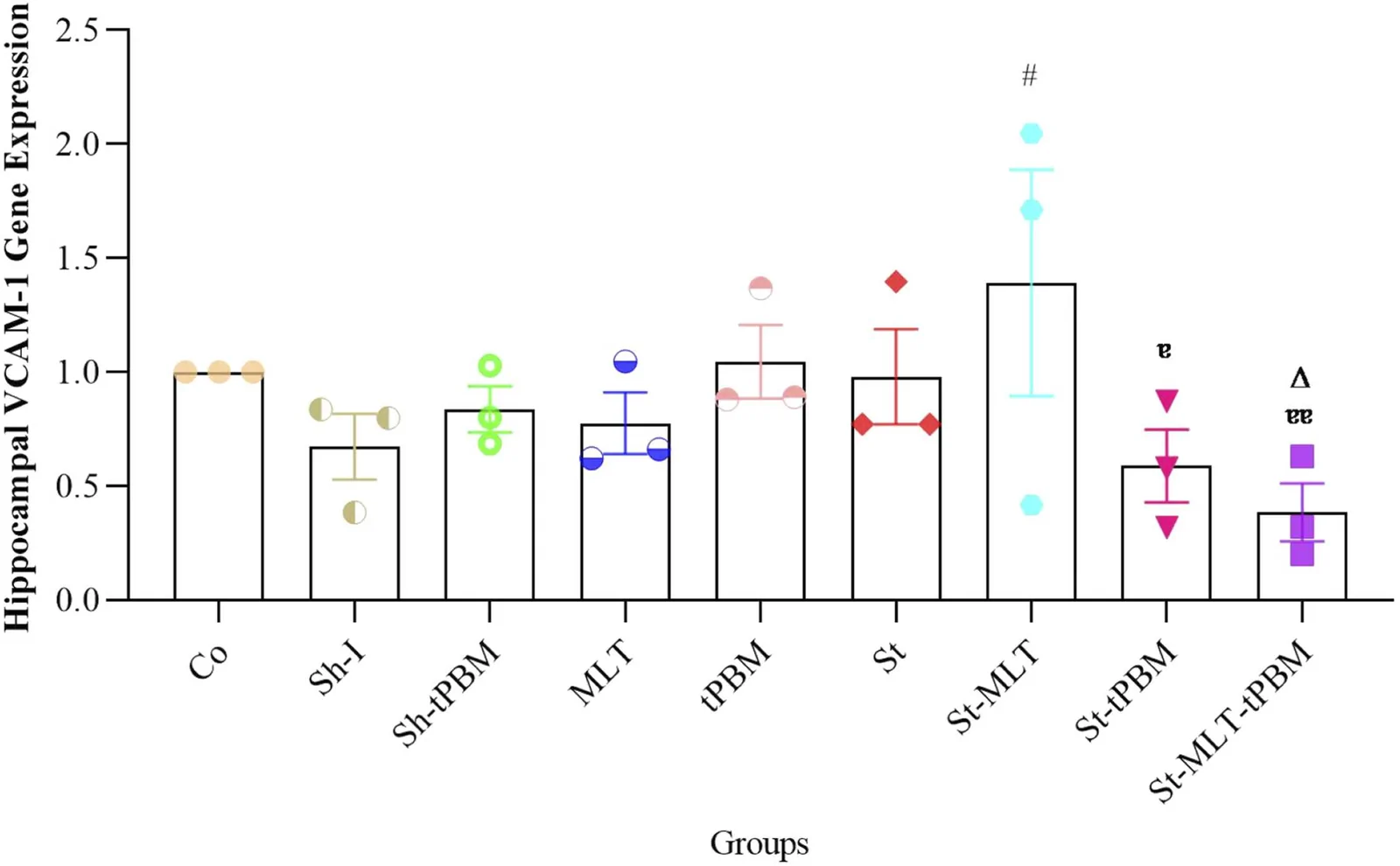
Hippocampal VCAM-1 gene expression measured by RT-qPCR (n = 3 independent biological replicates per group). The results are shown as the mean ± SEM (one-way ANOVA followed by LSD post hoc test). ^#^P < 0.05 compared to the Sh-I group. ^Δ^P < 0.05 compared to the tPBM group. ^ɐ^P < 0.05 and ^ɐɐ^p < 0.01 St^-^MLT group.

### 3.4 Hippocampal IL-1β gene expression

According to the data, there were no significant changes in the IL-1β gene expression among all groups (Fig. 5).

**Fig. 5:**
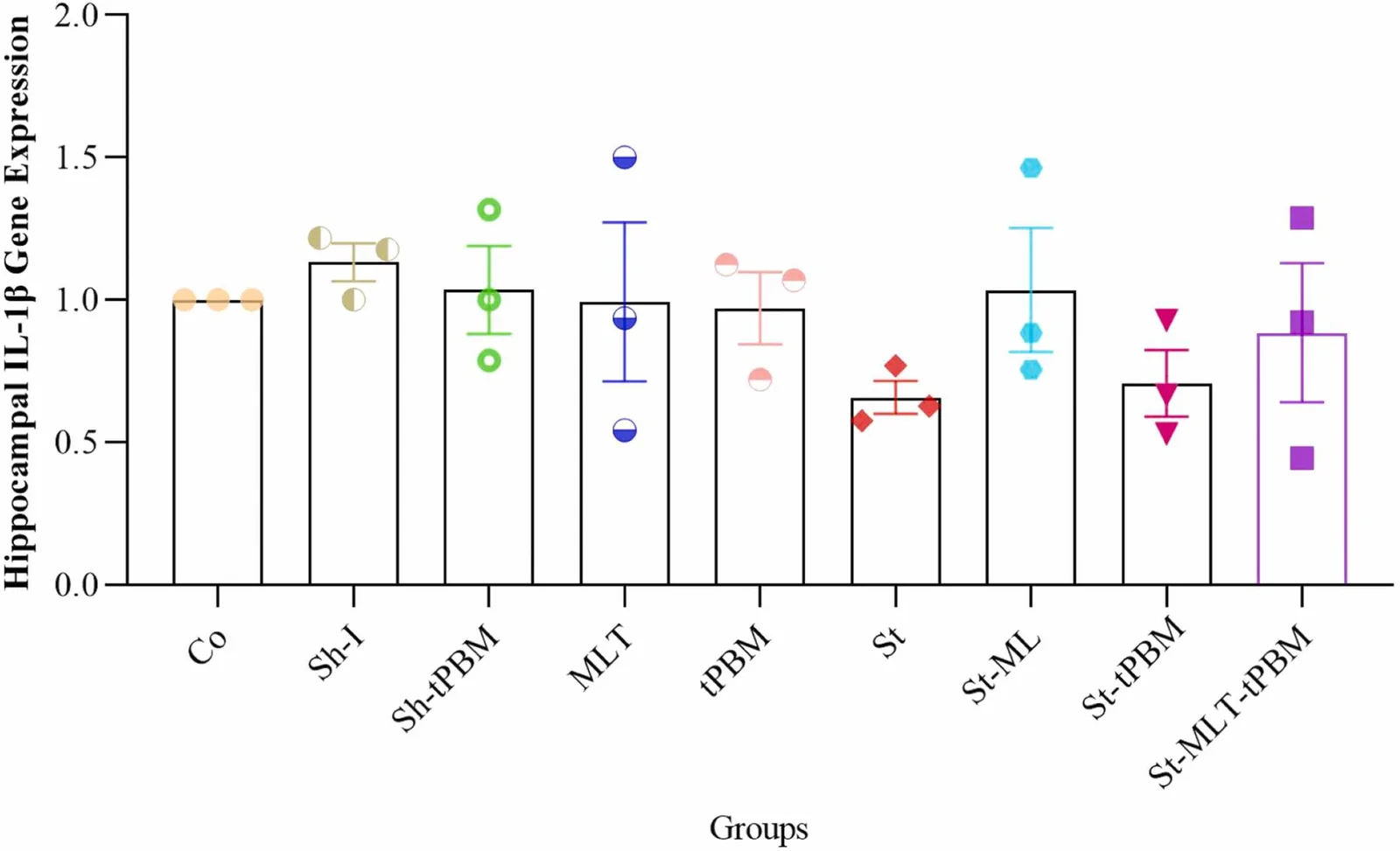
Hippocampal IL-1β gene expression measured by RT-PCR (n = 3 independent biological replicates per group). The results are shown as the mean ± SEM (one-way ANOVA followed by LSD post hoc test). There were no significant changes in the IL-1β gene expression among all groups.

### 3.5 Hippocampal Aif1 gene expression

Data showed that the hippocampal Aif1 gene expression was significantly decreased in the St group compared to all non-stressed groups (p < 0.01 for Co and Sh-tPBM; p < 0.001 for Sh-I and tPBM; p < 0.05 for MLT). However, MLT and combination of MLT and tPBM enhanced it in the stressed animals compared to the St group (p < 0.05 for St-MLT and p < 0.01 for St-MLT-tPBM). In addition, the Aif1 gene expression decreased in the St-MLT (p < 0.05) and St-tPBM (p < 0.01) groups compared to the tPBM group. Finally, it significantly decreased in the St-MLT-tPBM group compared to the Sh-I group (p < 0.05) (Fig. 6).

**Fig. 6:**
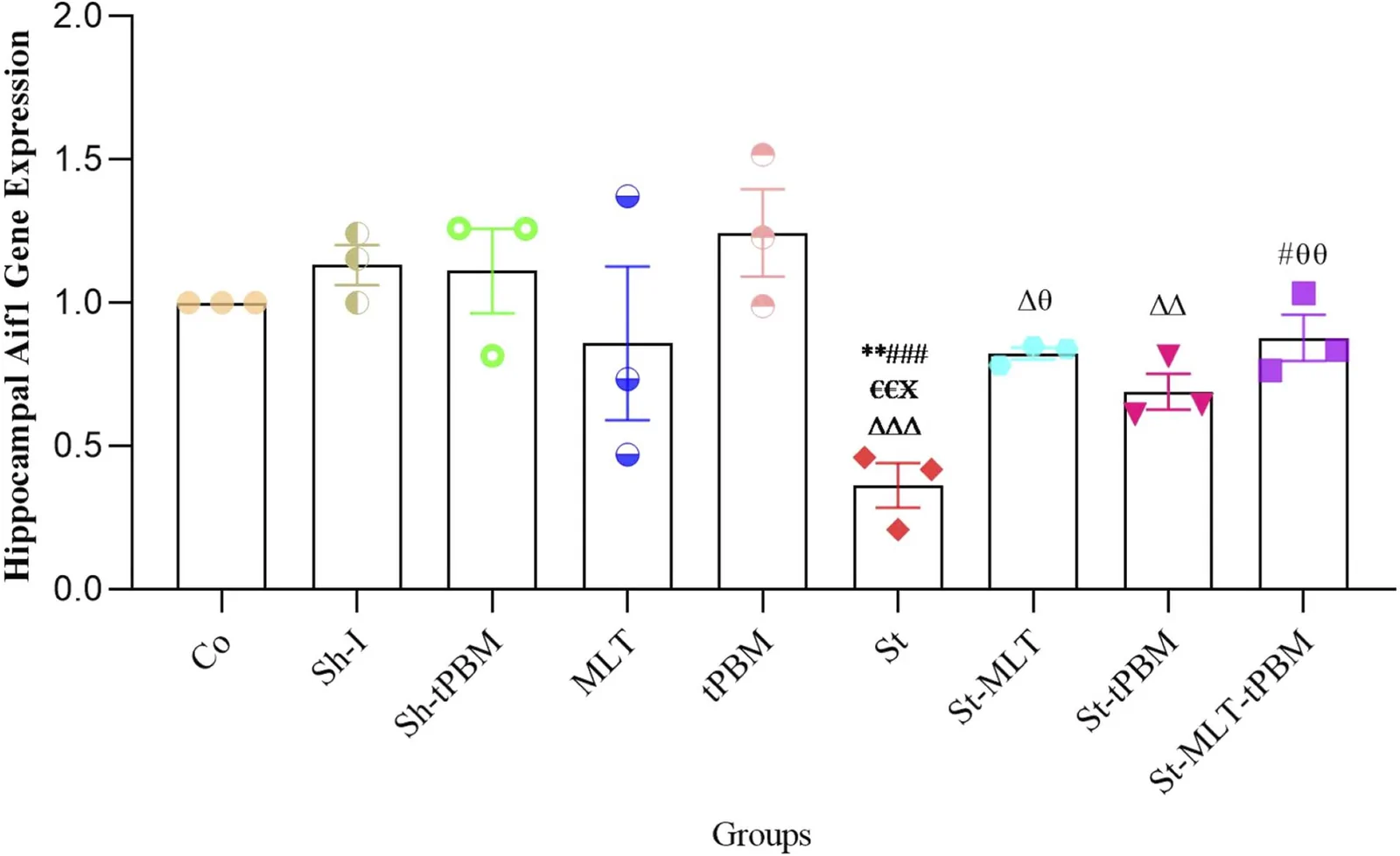
Hippocampal Aif1 gene expression measured by RT-PCR (n = 3 independent biological replicates per group). The results are shown as the mean ± SEM (one-way ANOVA followed by LSD post hoc test). **P < 0.01 compared to the Co group. ^#^P < 0.05 and ^###^p < 0.001 compared to the Sh-I group. ^€€^P < 0.01 compared to the Sh-tPBM^. Δ^P < 0.05, ^ΔΔ^p < 0.01, and ^ΔΔΔ^P < 0.001 compared to the tPBM group. ^θ^P < 0.05 and ^θθ^P < 0.01 compared to the St group.

### 3.6 EPM test

The Oat% was significantly decreased in the St group compared with all non-stressed groups (p < 0.01 in MLT and p < 0.05 for all other comparisons). Nevertheless, MLT and co-administration of MLT and tPBM increased Oat% in the Stressed subjects compared to the St group (P < 0.05 for St-MLT and p < 0.001 for St-MLT-tPBM). In addition, Oat% in the St-MLT-tPBM group was significantly enhanced compared to the St-tPBM group (p < 0.01) (Fig. 7).

**Fig. 7:**
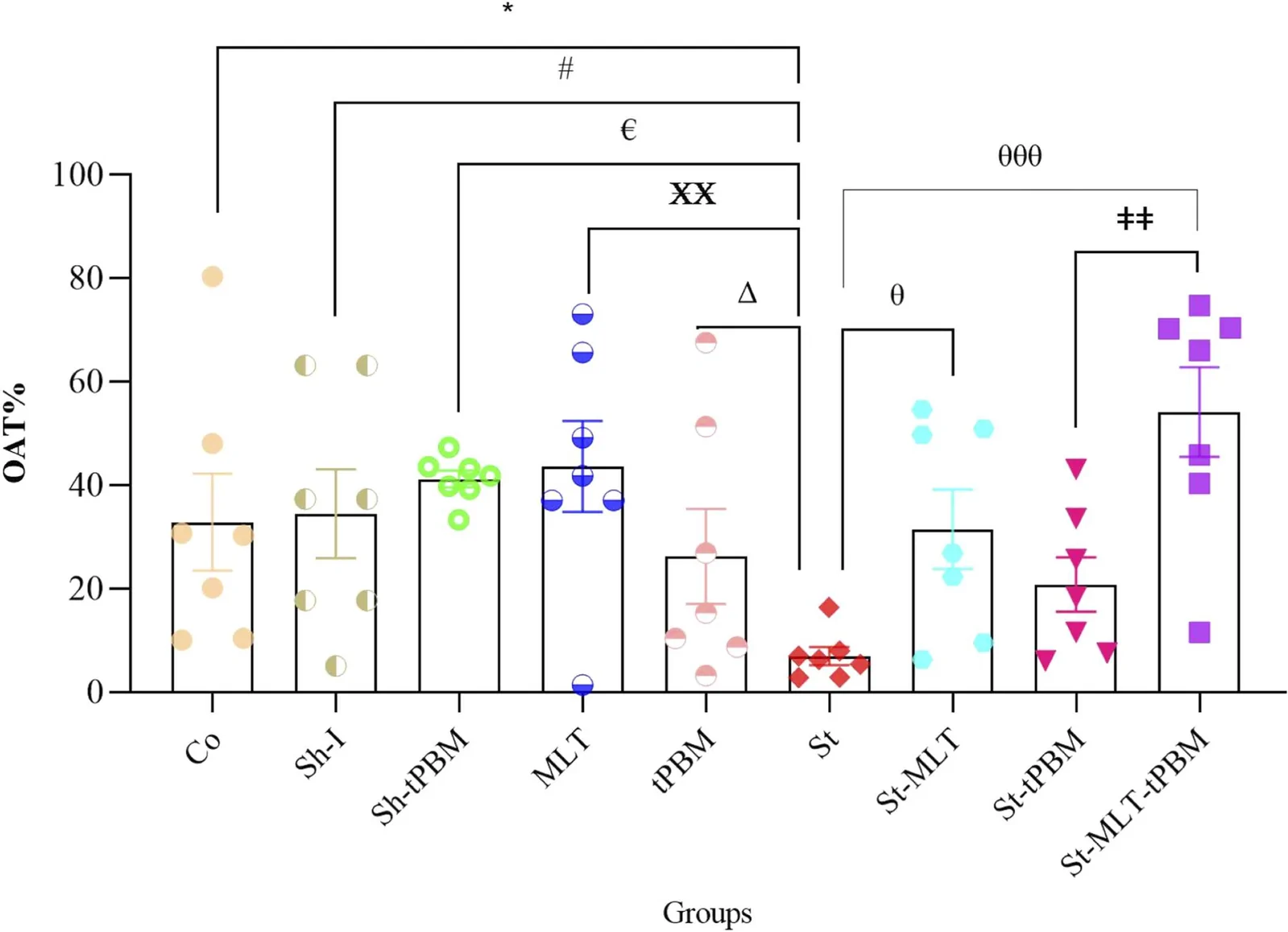
Percentage of time spent in the open arms (Oat%) during the EPM across groups (n = 7 per group). Results are reported as means ± standard error of the mean (one-way ANOVA, followed by LSD post hoc test). *p < 0.05 compared to the Co group; ^#^p < 0.05 compared to the Sh-I group; ^€^p < 0.05 compared to the Sh-tPBM; ^ӾӾ^p < 0.01 compared to the MLT group; ^Δ^P < 0.05 compared to the tPBM group; ^θ^p < 0.05 ^θθθ^p < 0.001 compared to the St group. ^ǂǂ^p < 0.01 compared to the St-tPBM group.

The OAE% was significantly decreased in the St group compared with all groups not exposed to stress (p < 0.05 in Sh-I and p < 0.01 in others). However, it was significantly increased in the St-MLT (p < 0.01) and St-MLT-tPBM (p < 0.001) groups compared with the St group. Finally, the OAE% was significantly increased in the St-MLT-tPBM group compared with the St-tPBM group (p < 0.05) (Fig. 8).

**Fig. 8:**
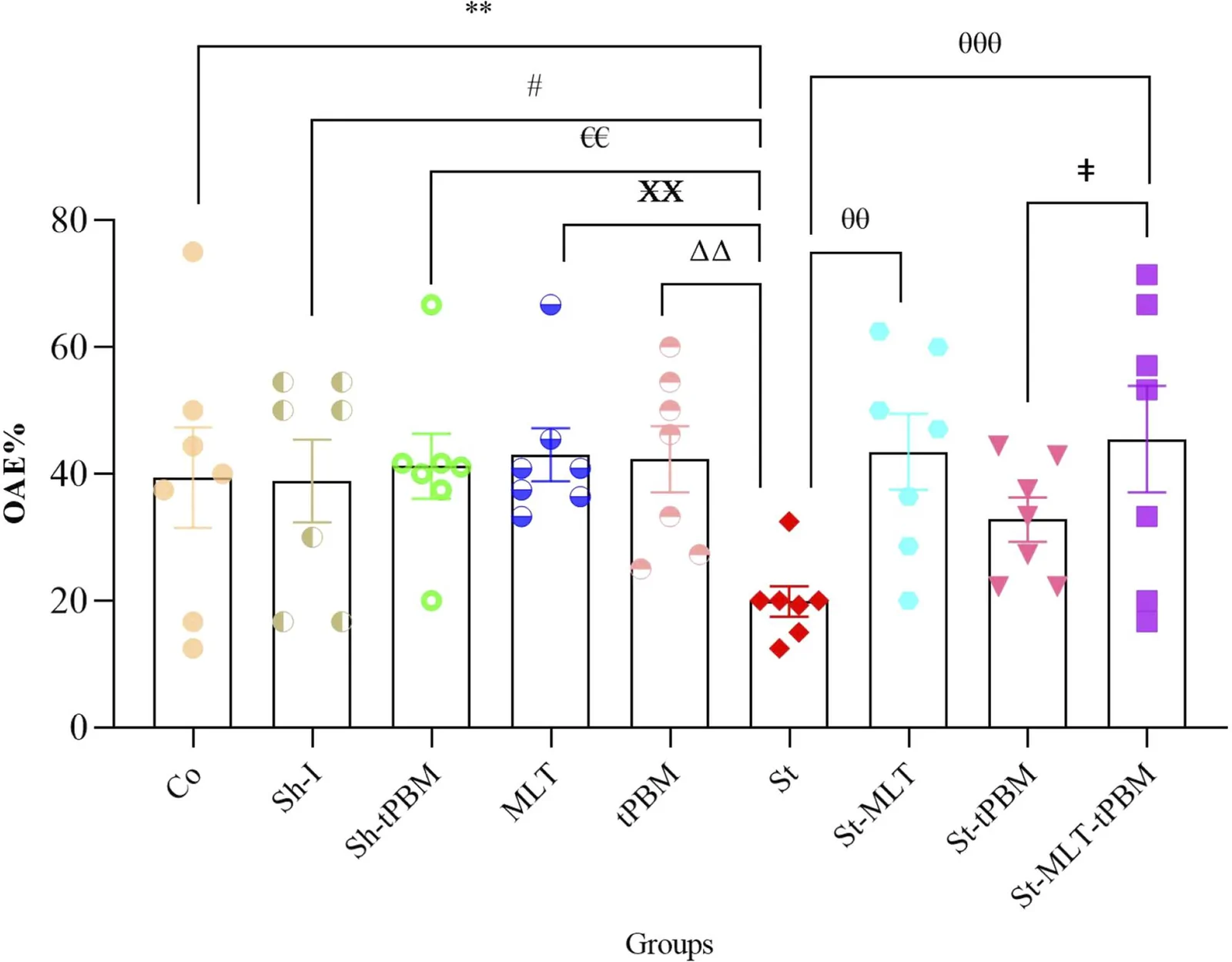
Percentage of open-arm entries (OAE%) during the EPM across groups (n = 7 per group). Results are reported as means ± standard error of the mean (one-way ANOVA, followed by LSD post hoc test). **p < 0.01 compared to the Co group; ^#^p < 0.05 compared to the Sh-I group; ^€€^p < 0.01 compared to the Sh-tPBM; ^ӾӾ^p < 0.01 compared to MLT group; ^Δ^P < 0.05 compared to the tPBM group; ^θ^p < 0.05 ^θθθ^p < 0.001 compared to the St group. ^ǂ^p < 0.05 compared to the St-tPBM group.

## 4 Discussion

This study investigated the effects of MLT and tPBM on the possible rehabilitation of neuroinflammation in chronically stressed rats by evaluating the gene expression levels of neuroinflammation-associated markers (IL-17A, ICAM- 1, VCAM-1, IL-1β, and Aif1), as well as their possible link with anxiety-like behaviors.

Chronic stress exposure for 21 days markedly increased hippocampal IL-17A gene expression, accompanied by significant upregulation of the adhesion molecule ICAM-1. Similarly, previous research showed that chronic stress noticeably increased IL-17A protein levels in the serum, hippocampus, and other brain regions of stressed rats (Peng et al., 2022b, Zhang et al., 2023). As a possible mechanism, a previous study reported that ICAM-1 contributes to T-cell polarization and facilitates T-cell crawling along the BBB endothelium (Abadier et al., 2015). The absence of ICAM-1 has been shown to significantly reduce both Th1- and Th17-mediated responses, highlighting the important role of CAMs in the development of neuroinflammation (Haghayegh Jahromi et al., 2020). The coordinated elevation of IL-17A and ICAM-1 suggests that stress could enhance pro-inflammatory cytokine signaling. Previous investigations reported that the expression of ICAM-1is also affected by IL-17A (Iwakura et al., 2008, Onishi and Gaffen, 2010). As a potential contributing factor, the endothelial cells between the blood and brain play a key role in leukocyte trafficking, mainly mediated by BBB structural components, including junctional molecules and CAMs (Barkaway et al., 2022, Namyen et al., 2020, Persidsky et al., 2006). In this regard, a previous investigation reported that rats under psychological stress showed enhanced mRNA expression of ICAM-1 and VCAM-1 in the hippocampus (Sawicki et al., 2015). These findings suggest a bidirectional relationship between IL-17A and CAMs. Chronic stress has been associated with increased recruitment of Th17 cells into the brain (Peng et al., 2022a), while IL-17A may further enhance the expression of adhesion molecules, such as ICAM-1 and VCAM-1, potentially contributing to immune cell recruitment (Wojkowska et al., 2017a). Another previous study has suggested a possible underlying process whereby stress-primed small intestinal γδ T cells (a distinct subset of T cells) can contribute to hippocampal mitochondrial dysfunction and depression-like behaviors through the production of IL-17A (Valter et al., 2024). However, it has been reported that chronic unpredictable stress over 8 consecutive weeks significantly decreases serum levels of IL-17A and IL-17F, while increasing IL-1β in mice (Shi et al., 2022a). In addition, although the IL-17A gene expression was reduced significantly in the St-MLT compared with the St group, it remained significantly higher than that of the normal subjects. Melatonin produced the opposite effect on CD4 + T cells, which depended on their activation status (Kuklina et al., 2016). MLT has been found to have either pro- or anti-inflammatory effects, depending on the systemic condition (Carrillo-Vico et al., 2013, Won et al., 2021). MLT has an opposite influence on Th17 cells (depending on their activation status), and the absence of a direct dose-response relationship for these effects is probably associated with a simultaneous expression of several types of its specific receptors by lymphocytes (Kuklina et al., 2016). It was suggested that the anti-inflammatory effects involve multiple signaling pathways, including antioxidant activity, inhibition of pro-inflammatory signaling, and regulation of inflammatory enzymes and transcription factors (Hardeland, 2018). Present findings demonstrated that MLT could not reverse the enhancement of cell adhesion molecule gene expression, especially ICAM-1, in the stressed rats, which could explain the heightening of IL-17A gene expression compared to normal subjects. Nevertheless, oral administration of melatonin at 5 mg/kg significantly reduced expression of ICAM-1 in the spinal cord of the rats (Kang et al., 2001). On the other hand, IL-17A and cell adhesion molecule gene expression were markedly decreased in the St-tPBM compared to the St groups. It seems that tPBM can restore gene expression levels of this important component of hippocampal immune response. tPBM has been reported to enhance mitochondrial function, particularly in cells that are metabolically compromised or under pathological stress (Dompe et al., 2020). By stimulating cytochrome c oxidase activity, tPBM increases ATP production, which subsequently activates multiple intracellular signaling pathways (Hamblin, 2017). Restoring cellular bioenergetics may help regulate inflammatory pathways, reduce neuroinflammation, and decrease apoptosis by influencing downstream redox-sensitive and cell survival signaling networks (Abijo et al., 2023, Nairuz and Lee, 2024). However, a prior study showed that tPBM could increase serum levels of IL-17A gene expression in non-stressed rodents and could not decrease it in the lipopolysaccharide-induced inflammation (Shamloo et al., 2023). Photobiomodulation has a bidirectional (pro- or anti-inflammatory) effect on inflammation, depending on the immune state of the target organism and the presence of inflammatory stimulants (Hu et al., 2024, Yang et al., 2025).

Different effects of tPBM on the IL-17A levels may refer to the diverse origins of inflammation. In general, previous investigations reported that tPBM could often ameliorate chronic stress-induced pro-inflammatory cytokines (Salehpour et al., 2019). According to the current data, the combination of MLT and tPBM produced a similar attenuation to that observed with tPBM on hippocampal IL-17A gene expression compared to rats experiencing chronic stress. This pattern suggests a more prominent contribution of tPBM, either alone or in combination with melatonin, to the normalization of hippocampal IL-17A expression following chronic stress. Some previous studies showed that the combination of tPBM with other safe treatments, like enriched environment and coenzyme Q10, had synergistic effects in rehabilitating brain functions in stressed rodents (Farazi et al., 2022, Salehpour et al., 2019).

In non-stressed animals, melatonin alone did not substantially alter IL-17A, ICAM-1, or VCAM-1 levels, indicating that these interventions do not provoke inflammatory activation under basal conditions. There was a difference in the effects of tPBM administration on ICAM-1 gene expression between stressed and non-stressed animals. Although tPBM increased ICAM-1 expression in non-stressed animals, it attenuated the stress-associated increase in stressed animals. Thus, the present findings do not support a simple anti-inflammatory effect of tPBM. Instead, they suggest a context-dependent effect of tPBM on immune-related signaling, which may vary according to the underlying physiological state (Hu et al., 2024), with its impact differing under basal and stress-related conditions. Further studies are required to determine the mechanisms underlying this context-dependent response. A previous study has further supported these findings by reporting increased serum and hippocampal IL-17A concentration following chronic stress (White et al., 2024). As a putative mechanism, prior research also reported that photobiomodulation therapy improves vascular function and decreases ICAM-1 expression by reducing NFκB activity (Junkoski et al., 2025, Nejatifard et al., 2021, Valter et al., 2024). On the other hand, the combination of MLT and tPBM attenuated the stress-induced upregulation of IL-17A gene expression and was similarly effective in reducing the stress-induced upregulation of ICAM-1 and VCAM-1 gene expression, particularly compared with MLT alone in stressed animals. Notably, both tPBM alone and the combined MLT and tPBM treatment produced a greater attenuation of the stress-associated increase in ICAM-1 and VCAM-1 expression than melatonin alone. However, the combined treatment did not show a significant advantage over tPBM alone, and no evidence of a synergistic effect was observed.

Aif1 expression was significantly reduced in the stress group; IL-1β levels remained unchanged, indicating no increase in the gene expression of these innate immune-related markers under the present stress conditions. Similarly, a previous study reported decreased Aif1 gene expression in a key brain region involved in the stress response following 14 consecutive days of chronic variable stress (Smith et al., 2016). It shows that Iba1-related gene expression and the number of microglial cells may vary depending on the type and duration of the stress regimen (Kopp et al., 2013, Smith et al., 2016). Although Aif1 encodes Iba1, a commonly used microglial marker, it has been suggested that its expression alone does not reliably indicate microglial activation or abundance (Guselnikova et al., 2026). Additionally, microglial responses may differ across brain regions and may not necessarily reflect a uniform microglial response throughout the brain (Lier et al., 2021). Thus, the observed reduction may reflect stress-associated changes in microglial gene expression or cellular composition rather than generalized changes in neuroinflammation-related gene expression. This interpretation is particularly relevant given the concomitant increases in IL-17A and ICAM-1 expression, suggesting that stress-related inflammatory responses may involve distinct and differentially regulated molecular pathways. The present findings suggest that 21 consecutive days of chronic restraint stress do not induce consistent changes in the expression of genes related to resident brain immune cell-associated inflammatory responses in the hippocampus. Consistent with current results, a previous study showed that chronic stress over 21 consecutive days did not elevate hippocampal or serum IL-1β protein levels in rats (Radahmadi et al., 2017). Conversely, chronic stress elevated hippocampal IL-1β protein levels after 28 (6 h/day) consecutive days (Rajput et al., 2017). It highlights the role of timing and stress intensity in the various immune responses. Mild to moderate stress does not typically elevate brain IL-1β (Barnard et al., 2019). It emphasizes the role of stress induction patterns in recruiting the type of immune responses. In contrast, stress markedly increased IL-17A and ICAM-1 expression, suggesting alterations in inflammatory and adhesion-related processes associated with Th17 responses and endothelial function. Given that IL-17A has been reported to modulate endothelial adhesion molecule expression, the concurrent elevation of IL-17A and ICAM-1 may reflect processes potentially related to immune cell trafficking across the BBB or changes in resident brain immune cell activity, although neither of these processes was directly assessed in the present study. It seems that Aif1 gene expression was not significantly increased in stressed rats because the peripheral immune responses preceded brain-resident immune cell activation (Peng et al., 2022b). However, this possibility was not directly assessed at the protein level in the present study. Stress duration is a key shared variable across protocols and should be carefully considered in study design. Pro-inflammatory cytokines such as TNF-α, IL-1β, and IL-6 are consistently upregulated in paradigms lasting three weeks or longer (White et al., 2024). Therefore, it seems that inducing chronic stress for 3 weeks was insufficient for immune response-related IL-1β release.

According to the EPM data, chronic stress resulted in marked decreases in Oat% and OAE% compared with those of normal subjects. This may suggest an association with the observed increase in IL-17A gene expression in stressed rats. The precise mechanisms underlying the relationship between inflammation and neuropsychiatric disorders remain to be elucidated; however, inflammatory responses may be associated with alterations in neurotransmitters and neural networks affected by psychological stress (Hassamal, 2023a). Previous research also reported that chronic psychological stress could decrease exploratory motivation and increase risk avoidance, as measured by the EPM test in rodent models (Mazaheri et al., 2024a, Liu et al., 2025). tPBM attenuated the stress-induced anxiety-like behavioral changes, resulting in EPM values that were no longer significantly different from those of the control group. However, these changes did not reach statistical significance compared with the stress group. tPBM significantly increased both the %OAT and %OAE when compared with the chronically stressed rodents (Meynaghizadeh-Zargar et al., 2020). Accumulating evidence indicates that tPBM may represent a promising approach for the treatment of brain disorders associated with neuroinflammation (Cardoso et al., 2022). In addition, melatonin ameliorated the anxiety-like behavior in the stressed subjects. MLT has shown anxiolytic-like effects in rodents in different behavioral paradigms of anxiety (Repova et al., 2022). Melatonin increased the time spent in the open arms of the EPM in healthy rats and pinealectomized rats (Ochoa-Sanchez et al., 2012, Bustamante-García et al., 2014). In this research, the anxiolytic effects of MLT did not consistently correlate with neuroinflammation-related gene expression, as IL-17A and ICAM-1 gene expression levels remained elevated in the stress-MLT group compared with the normal subjects. The protective effects of MLT against anxiety-like behaviors may involve other molecular markers and related pathways not examined in the present study. In this regard, evidence suggests that MLT possesses potent neuroprotective properties (Alghamdi, 2018) that promote cytoprotection by modulating oxidative stress, apoptosis, and mitochondrial homeostasis (Chrustek and Olszewska-Słonina, 2021, Yanar et al., 2019). MLT has been investigated as a premedication for alleviating preoperative and postoperative anxiety in humans. (Caumo et al., 2009). Studies showed that MLT supplementation enhances antioxidant defenses and reduces lipid peroxidation (Zarezadeh et al., 2022). The combination of MLT and tPBM significantly enhanced Oat% and OAE% compared with both the St and St-tPBM groups, suggesting that the combined intervention may provide greater anxiolytic effects than tPBM alone under chronic restraint stress.

This study has some limitations that should be highlighted. Although transcriptional changes provide valuable mechanistic insight, mRNA levels do not always directly correspond to protein abundance or functional activity due to post-transcriptional and post-translational regulatory mechanisms (Born et al., 2017). Therefore, future studies incorporating protein quantification approaches such as Western blotting or immunohistochemistry would provide a more comprehensive understanding of the inflammatory and microglial alterations observed. Moreover, as a protective intervention, MLT was administered during the day, 30 min after stress induction, with the same timing maintained across all groups receiving MLT or tPBM interventions for experimental consistency. However, although previous studies have administered MLT shortly after stress induction (Lee et al., 2018, Fenton-Navarro et al., 2021), nighttime administration has also been suggested to enhance its neuroprotective effects (Essawy et al., 2023, Estrada-Reyes et al., 2018, Haridas et al., 2013). Further studies are therefore warranted to determine the optimal timing of MLT administration to maximize its therapeutic efficacy, particularly in combination with other interventions. Another limitation of this study is the inclusion of male rats only, which may limit the generalizability of the findings to females (Bhargava et al., 2021). Future studies should include both sexes to assess potential sex-related differences in the responses to chronic stress and the interventions. Another limitation is that only a single dose of melatonin and a single set of tPBM parameters were evaluated. The absence of dose–response analyses limits conclusions regarding optimal therapeutic intensity and prevents the determination of whether greater or lower doses might yield differential or more pronounced effects. Applying tPBM therapy to brain regions other than the hippocampus may yield even greater improvements. Systematic evaluation of multiple dosages and stimulation parameters would strengthen translational relevance and clarify potential threshold or ceiling effects.

## 5 Conclusion

In the present study, chronic restraint stress was associated with increased expression of genes involved in inflammatory responses, particularly IL-17A and ICAM-1, in the hippocampus. This is associated with the generation of anxiety-like behaviors in stressed subjects. In contrast, MLT treatment in stressed rats reduced IL-17A gene expression compared with untreated stressed rats. MLT also reduced anxiety-like behaviors, which may be related to other neuroprotective effects of MLT that extend beyond the gene expression changes assessed in the present study. tPBM therapy also showed potential to counteract stress-associated increases in hippocampal IL-17A and ICAM-1 gene expression. In parallel, the attenuation of anxiety-like behavior extended to a level at which the observed behavioral measures no longer differed significantly from those of the control group. In non-stressed rats, tPBM increased ICAM-1 gene expression, which could generate considerations about utilizing this treatment in healthy subjects. Co-administration of MLT and tPBM showed a pattern of effects similar to tPBM alone for stress-induced changes in IL-17A and ICAM-1 gene expression, whereas its effects on anxiety-like behaviors mirrored those of MLT alone and were greater than those observed with tPBM alone. On the other hand, while IL-17A and ICAM-1 were upregulated, IL-1β remained unchanged, and Aif1 decreased, highlighting the potential importance of temporal dynamics in shaping neuroinflammation-related responses. Overall, this study supports the potential of MLT and tPBM as complementary approaches for mitigating stress-related alterations and underscores the need for further investigation to optimize their doses and treatment parameters.

## CRediT authorship contribution statement

**Mohammad Mazaheri:** Writing – review & editing, Writing – original draft, Visualization, Validation, Investigation, Data curation, Conceptualization. **Maryam Radahmadi:** Writing – review & editing, Formal analysis, Data curation. **Fahimeh Hosseini-Nasab:** Investigation, Data curation. **Nahid Eskandari:** Writing – review & editing, Visualization, Project administration, Formal analysis, Conceptualization.

## Ethics declaration

This study was conducted in accordance with the ARRIVE (Animal Research: Reporting of In Vivo Experiments) guidelines. This study was approved by the Research Ethics Committees of Laboratory Animals - Isfahan University of Medical Sciences. (Approval No. IR.MUI.AEC.1403.065)

## Declaration of Competing Interest

The authors declare that they have no known competing financial interests or personal relationships that could have appeared to influence the work reported in this paper.
