# Supplementary material for: Effects of melatonin and transcranial photobiomodulation on neuroinflammation-related gene expression and anxiety-like behaviors in rats under chronic restraint stress
Source: IBRO Neurosci Rep. 2026 Oct 18;21:1064–76. doi: 10.1016/j.ibneur.2026.10.009 (PMC13743380; doi:10.1016/j.ibneur.2026.10.009)
Supplement: Supplementary file 1 [file mmc1.docx]

**Supplementary Figure**

Supplementary Figure 1. RT-qPCR melting curves. Representative melting curves obtained from the RT-qPCR assays for IL-17A (A), ICAM-1 (B), VCAM-1 (C), Aif1 (D), IL-1β (E), and GAPDH (F) are shown. The melting curves were examined to assess the specificity of the amplified products.
